# Obesity and the burden of health risks among the elderly in Ghana: A population study

**DOI:** 10.1371/journal.pone.0186947

**Published:** 2017-11-08

**Authors:** Godfred O. Boateng, Ellis A. Adams, Mavis Odei Boateng, Isaac N. Luginaah, Mary-Margaret Taabazuing

**Affiliations:** 1 Department of Anthropology & Global Health, Northwestern University, Evanston, Illinois, United States of America; 2 Global Studies Institute, Georgia State University, Atlanta, Georgia, United States of America; 3 Department of Geosciences, Georgia State University, Atlanta, Georgia, United States of America; 4 Department of Social Work, University of Windsor, Windsor, Ontario, Canada; 5 Department of Geography, Western University, London, Ontario, Canada; 6 Department of Medicine, Division of Geriatric Medicine, Western University, London, Ontario, Canada; 7 Department of Medicine, London Health Sciences Centre, Victoria Campus, London, Ontario, Canada; Dasman Diabetes Institute, KUWAIT

## Abstract

**Background:**

The causes and health risks associated with obesity in young people have been extensively documented, but elderly obesity is less well understood, especially in sub-Saharan Africa. This study examines the relationship between obesity and the risk of chronic diseases, cognitive impairment, and functional disability among the elderly in Ghana. It highlights the social and cultural dimensions of elderly obesity and discusses the implications of related health risks using a socio-ecological model.

**Methodology:**

We used data from wave 1 of the Ghana Study on Global Ageing and Adult Health (SAGE) survey-2007/8, with a restricted sample of 2,091 for those 65 years and older. Using random effects multinomial, ordered, and binary logit models, we examined the relationship between obesity and the risk of stage 1 and stage 2 hypertension, arthritis, difficulties with recall and learning new tasks, and deficiencies with activities of daily living and instrumental activities of daily living.

**Findings:**

Elderly Ghanaians who were overweight and obese had a higher risk of stage 1 and stage 2 hypertension, and were more likely to be diagnosed with arthritis and report severe deficiencies with instrumental activities of daily living. Those who were underweight were 1.71 times more likely to report severe difficulties with activities of daily living. A sub analysis using waist circumference as a measure of body fat showed elderly females with abdominal adiposity were relatively more likely to have stage 2 hypertension.

**Conclusions:**

These findings call for urgent policy initiatives geared towards reducing obesity among working adults given the potentially detrimental consequences in late adulthood. Future research should explore the gendered pathways leading to health disadvantages among Ghanaian women in late adulthood.

## Introduction

Health risks mediating the relationship between aging and morbidity or mortality fall broadly into chronic and cardiovascular-related diseases (hypertension, angina, arthritis, stroke, and diabetes), cognitive impairments, difficulties with Activities of Daily Living (ADLs) or functional disabilities, depression, osteoporosis, sleep disorders, vision changes, and hearing impairments [1, 2]. Among these health risks, cardiovascular diseases alone contribute up to 30.3% of the total burden of diseases among the elderly; while chronic respiratory diseases account for 9.5%; and neurological and mental disorders make up 6.6% [3]. These multiple risk factors associated with cardiovascular diseases are reinforced by being overweight or obese [4]. Previously more prevalent in high-income countries, cardiovascular diseases and associated mortality rates are increasing rapidly in developing countries due to growing rates of obesity [2].

Obesity is no longer prevalent only among young people; obesity among the elderly is also increasing globally and has significant public health consequences [5–8]. Although the effects of obesity on mortality and morbidity are well-studied and do point to diabetes and cardiovascular illnesses as common mediating risk factors [9–11], the effects on elderly populations could be more devastating. The impacts of elderly obesity include heart failure, impaired physical functionality, arthritis, cancers, and hypertension [7, 12–14]. Elderly obesity also increases the risk of dementia [15] and diabetes [16].

Despite increasing trends in elderly obesity in developing countries, less attention has been devoted to understanding the associated health risks. Studies on obesity in sub-Saharan Africa (SSA) have tended to focus on younger populations to the neglect of older adults (>50 years) [17]. The scanty scholarship that focuses on obesity in SSA shows that obese adults may be prone to cardiovascular and respiratory diseases, liver malfunctions, and diabetes [18]. It remains unclear whether and how obesity may facilitate other health risks among the elderly in SSA. Using Ghana as a case study, we contribute to the literature in this regard. We examine the relationship between obesity and the burden of health risks (i.e. chronic diseases, cognitive impairment, and functional disability) among the elderly, as well as assess the associating effects of social determinants of health.

### Health risks associated with elderly obesity

The health risks associated with obesity are too numerous for our paper to sufficiently engage. Consequently, we limit our focus to two chronic diseases (hypertension and arthritis), cognitive impairment and functional disability. These health risks were selected because of their increasing prevalence among the elderly in SSA [19, 20].

#### Chronic diseases

Nearly 80 percent of global mortalities associated with chronic diseases occur in low and middle-income countries [2, 21]. Among the chronic diseases, cardiovascular conditions such as hypertension are a leading cause and the epidemiological evidence suggests a strong association with obesity. Dorresteijn et al. [22] note that overweight and obesity often lead to adipose tissue dysfunction, which may also result in enlarged hypertrophied adipocytes and significant changes in the secretion of adipokines and free fatty acids in the body. They also observe that chronic vascular inflammation, oxidative stress, activation of renin-angiotensin-aldosterone system and sympathetic overdrive due to overweight and obesity eventually predispose one to hypertension. The combined effect of obesity and hypertension subjects the elderly to far more severe health risks [23]. Further, obesity, hypertension, and diabetes often coexist and worsen health-related quality of life [24]. While hypertension progresses in stages, most studies on chronic conditions do not differentiate between the different stages. We address this common omission in our study by accounting for both stage 1 and stage 2 hypertension in our analysis.

Another chronic condition we examine is arthritis. Elsewhere, arthritis is noted as one of the leading causes of disability [25, 26]. Recent studies show that 49.6% of persons aged 65 years or older in the United States have ever reported doctor-diagnosed arthritis. Despite the evidence showing an association between obesity and arthritis, few studies have examined the relationship among the elderly in SSA in part due to scarcity of data on the prevalence of arthritis in the region [27]. Osteoarthritis, a common form of arthritis in Africa, is often associated with severe degeneration of joints including the knee and the hip, and chronic disability among the elderly [28, 29]. In older adults, the incidence of osteoarthritis is strongly linked to obesity [30–32], and noted to cause morbidity and mortality [11]. Obesity exerts an increased mechanical stress on the tibiofemoral cartilage and produces inflammation through adipose tissues, which orchestrates the pathophysiological processes leading to osteoarthritis [29].

#### Cognitive impairment

Obesity is associated with cognitive impairment among young adults and adolescents [33]. Miller and Spencer [34] indicated that “obesity-associated systemic inflammation leads to inflammation within the brain, particularly the hypothalamus, resulting in partial negative cognitive outcomes.” However, among the elderly, the evidence linking elderly obesity to cognitive impairment remains equivocal due to an uncertain relationship between body-fat content and ageing [35]. While some studies show that obesity in older adults may lead to cognitive decline [36], others conclude that the relationship may be indirect, or that obesity may actually boost cognitive function [37]. Few studies examine the relationship between obesity and cognitive function among the elderly in developing regions. For example, Amarya et al. [5] engage marginally with the effects or functional implications of obesity without explicitly analyzing the relationship. The rising rates of obesity among older adults in developing regions necessitate scholarship to better understand its potential role in cognitive impairment.

#### Functional disability

Obesity may be a strong predictor of declining physical functionality in older adults [38, 39]. In older adults, obesity limits physical functionality primarily through arthritis [28, 40], but also through decreased metabolism that affects the performance of daily activities [41, 42]. Alternatively, declines in physical functionality may be associated with age-related declines in muscle mass that become more rapid with advanced age. For example, Baumgartner et al. [43] discovered among an elderly population in New Mexico that *sarcopenia*, defined as a low relative muscle mass, is a significant predictor of self-reported physical disability. Thus, limited functionality, while not always a direct attribute of obesity, may still have an indirect role in facilitating sarcopenia in older adults [44]. Despite the evidence linking obesity among the elderly to functional disability, rarely has any study tested this relationship in SSA.

### An ecological model on the effects of health risks

**Fig 1:**
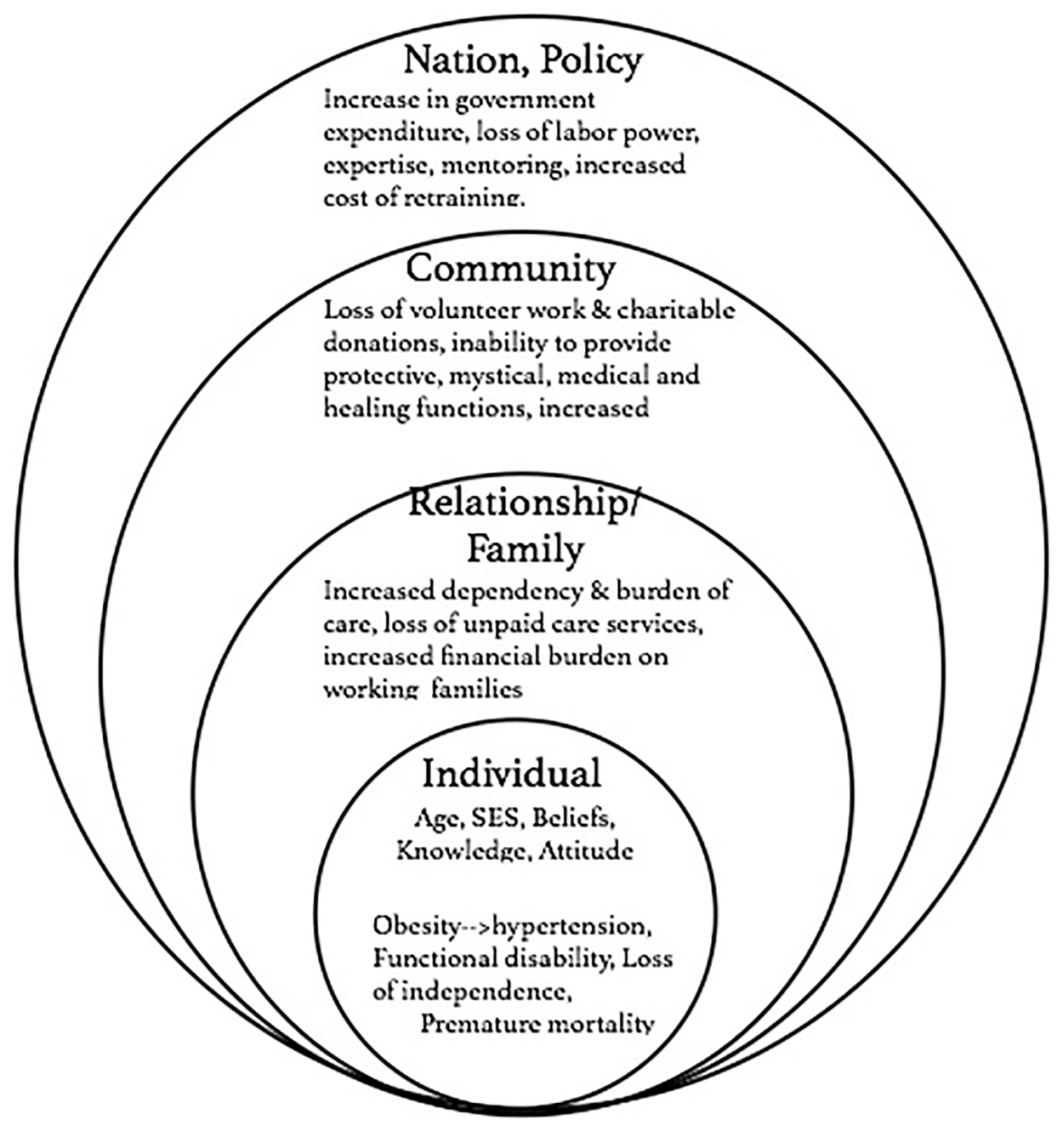
The ecological model: A framework for understanding the effects of health risks among the elderly.

We adapted Bronfenbrenner’s 1977 ecological model [45] to explain the relationship between obesity and other health risks among the elderly. The ecological model (also known as socio-ecological model) examines health outcomes, behavior, and risks based on the social and physical environment (Fig 1). The model suggests that health risks and outcomes can be understood as byproducts of the multiple interactions between society and environmental factors, suggesting that different environmental and social factors codetermine health outcomes. In this study, we develop a variation of the ecological model to explicate the multilevel influences of hypertension, arthritis, cognitive impairment, and functional disabilities on individuals, families, communities, and societies/countries. The nested rings in the model explain how the influence of health risks at one level affects other levels.

The first level highlights the effects of health risks such as hypertension, arthritis, cognitive impairment and deficiencies in activities of daily living on the individual. Often, the individual’s age, socio-economic status, ethnicity, knowledge, attitude, and beliefs could exacerbate health risks. Elderly people who suffer from hypertension or arthritis are at a greater risk of experiencing other comorbidities and increased risk of premature mortality [46]. Functional disability among the elderly is accompanied by the loss of independence, i.e. their inability to perform basic activities of daily living, engage in social relationships and keep good health [47, 48].

The second level identifies the effects of these health risks on close relationships. The elderly generally depend on social networks, especially nuclear and extended family members and friends to help with daily activities. This dependency is worsened if the elderly are chronically sick or disabled, which increases the burden of care for families [47, 48]. Subsequently, family members who serve as caretakers may suffer from stress, depression, anxiety, financial loss, vicarious trauma, and at times, similar health risks. The situation is more precarious in most developing countries where a majority of the elderly do not have health insurance to access health care; where health insurance is available, coverage favors wealthier classes of people [49].

The elderly are not only recipients of care; they also provide unpaid care services to their families. Many serve as caregivers to their grandchildren, relatives, friends and neighbors, with women playing a greater role in this function than men. Increase in health risks weakens their ability to provide such services, increasing the financial burden on working families as they outsource this responsibility to schools and professional caregivers.

The third level identifies the effects of these health risks on the community. The elderly have a wealth of skills and experiences, and contribute significantly to volunteer work and charitable donations. In most African countries, the elders contribute to the socialization and education of the young, play protective, mystical, medical and healing functions, provide economic support to households/communities, and maintain societal values and norms [50–52]. They are a great resource of knowledge and experience to the younger generation. A decline in their health results in both cultural and financial losses to the community. The increasing health risks among the elderly also means an overdependence on community services.

The cost of these health risks at the national level is also quite significant as it can increase government expenditure on health to the detriment of other sectors [53]. It could also lead to budget deficits as governments allocate more resources to manage the health of the elderly. Economically, the loss of labor power, the expertise of the elderly, and their mentoring due to early mortality will increase the cost of retraining for most governmental institutions. Consequently, policies and laws at the local and national level are needed to mitigate the effects of obesity and promote healthy lifestyles.

In the case of Ghana, the probability of dying between the ages of 30 and 70 years for individuals suffering from non-communicable diseases is currently estimated at 20% [54]. The effect may be higher for those over 70 years and obese. The burden of care on families’ due to elderly obesity and related effects is underpinned by cultural and religious values. Not caring for the elderly may come with ridicule, ostracism, guilt, or stigma. These cultural and social consequences compel families to take on aging relatives, which comes with financial costs as well as emotional and psychosocial drain. The lack of residential care facilities for the elderly also leaves the burden of care on young working professionals, which may impact their productivity and wellbeing.

Collectively, these negative effects make it crucial to understand the factors associated with the health risks among the elderly in Ghana and elsewhere in SSA. The biomedical evidence linking obesity to health risks seems well understood. What has not been sufficiently explored are the relationships between obesity and health risks among the elderly and what role—if any—is played by social determinants. To advance new knowledge to fill these gaps, our study addresses six distinct but interrelated hypotheses:

1. The expected risk for obese vs. normal weight elderly respondents will be greater for those experiencing stage 1 or stage 2 hypertension instead of normal/pre-hypertension.
2. Obese elderly will more likely experience arthritis than those with normal weight.
3. Obese elderly will more likely experience severe difficulties with recall than those with normal weight.
4. Obese elderly will more likely experience severe difficulties with learning new tasks than those with normal weight.
5. Obese elderly will more likely experience severe deficiencies with activities of daily living than those with normal weight.
6. Obese elderly will more likely experience severe deficiencies with instrumental activities of daily living, which are activities that enable the elderly to retain independence.

## Methods

### Study context

Ghana’s age structure shows 9% of the population are 55 years and above, with 34.05% between 25 and 54 years old [55]. While the age structure suggests Ghana’s population is relatively young, the ageing cohort has been increasing gradually since 2000 [56]. The WHO [20] assessment report on ageing and health in Ghana show that the number of elderly people in Ghana has increased more than seven-fold between 1960 and 2010; growth in this ageing population has outpaced socioeconomic development. With increase in life expectancy, improved public health measures, better nutrition and personal hygiene, and declining fertility, the ageing population will only increase in subsequent years [19]. Accompanying this ageing trend is the increased prevalence of obesity in older adults. Ofori-Asenso et al. [57] show that 43% of Ghanaian adults are either overweight or obese, with obesity significantly higher in women than in men.

### Data description

We used data from Wave 1 of the Study on Global Ageing and Adult Health (SAGE) Survey collected in Ghana from May 2007 to June 2008 through collaboration between the World Health Organization (WHO), the Ghana Ministry of Health and the University of Ghana Medical School. SAGE Wave 1 is a nationally representative multi-country study conducted as part of a longitudinal survey program by the WHO to monitor the health and well-being of adult populations aged 50 years and above in Ghana, China, India, Mexico, the Russian Federation and South Africa, between 2007 and 2010 [58]. For comparative purposes, SAGE focused on two target populations: a large sample of persons aged 50 years and older and a smaller sample of persons aged 18–49 years. For the survey in Ghana, a stratified multistage cluster design was used to select respondents. The sample was stratified by region (all ten administrative regions) and location (urban/rural). A sample of 5000 respondents 50 years and older and 1000 participants between the ages of 18–49 years were interviewed. This required the use of 250 Enumeration Areas (EA) out of the 298 EAs used during SAGE Wave 0. Twenty (20) households which had one or more 50 or older individuals, and four households with members aged 18–49, were selected from each EA. Both household and individual questionnaires were administered to selected respondents. Response rates at both household and individual levels were 86% and 80%, respectively. Respondents were asked questions about their household and related characteristics, risk factors, anthropometry, preventive health behaviors, chronic conditions, and blood pressure [59]. With a focus on the elderly, we restricted our analysis to only respondents aged 65 years and above in the individual dataset leading to an analytical sample of 2,091.

### Outcome variables

In this analysis, we focus on 6 outcome variables. Based on the availability of data, we examined hypertension and arthritis for chronic diseases, difficulties with recall and performing new task for cognitive impairment, and deficiencies with Activities of Daily living (ADLs) and Instrumental Activities of Daily Living (IADLs) for functional disability.

#### Hypertension

The measure of hypertension was taken from objective measurements of respondents’ blood pressure (systolic/diastolic), which were taken 3 times. The mean of all three readings was used as a diagnosis for hypertension. Most studies have used the binary category of systolic blood pressure ≥140 mmHg and/or diastolic ≥90 mmHg [1, 59]; however, this form of grouping does not differentiate between the stages of hypertension as recommended by the American Heart Association, making it difficult for proper interventions to be put in place to mediate unhealthy blood pressure ranges. The American Heart Association recognizes five blood pressure ranges: normal blood pressure (<120/80 mm Hg), prehypertension (120-139/80-89 mm Hg.), stage 1 hypertension (140-159/90-99 mm Hg.), stage 2 hypertension (≥160/100mm Hg.) and hypertensive crisis (>180/110 mm Hg) [60]. At stage 1, doctors are more likely to prescribe lifestyle modifications, but at stage 2, doctors will prescribe a combination of blood pressure medication and lifestyle changes. With less than 10 percent of our sample in the hypertensive crisis category, we merged the categories into three to differentiate between hypertension stage 1 and 2, against normal/prehypertension. Systolic blood pressure readings of <140mmHg/diastolic blood pressure <90 mmHg was classified as normal/prehypertensive (0), systolic blood pressure readings of 140–159 mmHg/diastolic 90–99 mmHg was classified as stage 1 hypertension (1), and systolic blood pressure readings ≥160mmHg/diastolic ≥100 was classified as stage 2 hypertension (2).

#### Arthritis

For arthritis, respondents were asked whether they had ever been diagnosed with/told they had arthritis (a disease of the joints, or by other names rheumatism or osteoarthritis). The response was coded as (0) for no, and (1) for yes. The variable was therefore measured as a binary.

#### Cognitive impairment

A measure of respondent’s cognitive impairment was created from the following questions: overall in the last 30 days, how much difficulty did you have with concentrating or remembering things? The second question asked: overall in the last 30 days, how much difficulty did you have in learning a new task (for example, learning how to get to a new place, learning a new game, learning a new recipe)? The responses to both questions were scored on a 5-point Likert type scale ranging from 1 (none) to 5 (extremely difficult/cannot do). A preliminary analysis revealed a response rate of <2% for the ‘extreme’ response categories. Consequently, we merged severe and extreme categories together resulting in four response categories coded as (1) none (2) mild (3) moderate and (4) severe/extreme. Both variables were measured at the ordinal level.

#### Functional disability

To make functional assessments of the respondents, we used SAGE questions on ADLs and IADLs. The 17 items making up ADLs included questions about difficulties in the past 30 days with sitting for long periods, walking 100 meters, standing up from sitting down, climbing one flight of stairs without resting, washing your whole body, getting up from lying down, getting to and using the toilet. This constituted a Cronbach alpha of 0.87. The IADLs consisted of 5 questions which asked about difficulties in the past 30 days with taking care of household responsibilities, joining in community activities, day to day work, getting where you want to go, using private or public transport if needed, and getting out of your home. The Cronbach’s alpha for these five items was 0.85. The responses to these questions were scored using a 5-point Likert type scale as (1) none (2) mild (3) moderate, (4) severe, and (5) extreme. These response categories were then dichotomized into a binary category: (0) no deficiency consisting of 1–3 and (1) severe deficiency consisting of 4–5. Both variables were measured at the binary level.

### Independent variables

#### Obesity

The main independent variable examined in this paper was obesity. BMI, an index of relative weight (in Kilograms) divided by the square of respondents’ height in meters (kg/ m^2^), was used to assess obesity. The measurement of height and weight in this data followed a standard methodology [61]. Calculated BMI scores were recoded based on WHO’s international classification of adult underweight, overweight and obesity into a nominal variable consisting of (1) normal = 18.50–24.99 kg/m^2^, (2) underweight<18.50 kg/m^2^, (3) overweight = 25–29.99 kg/m^2^, and (4) obese≥30 kg/m^2^ [62, 63]. The use of only BMI may limit our ability to capture visceral adiposity. Waist circumference (WC) has been found to be a better predictor of intra-abdominal adipose tissue and hypertension than BMI [64]. Consequently, to confirm the BMI results on hypertension, we conducted a sub-analysis following recommended WC thresholds for increased cardio metabolic risk in men (>102cm) and women (>88cm) [64].

We then adjusted for co-morbidities (angina and diabetes) and the social determinants of health [65]. This included psychosocial factors (depression and happiness), behavioral/lifestyle factors (alcohol consumption and vigorous activities), socio-cultural (education, wealth, employment, religion, and place of residence), and biosocial (sex and ethnicity) factors.

#### Co-morbidities

Angina and diabetes have been found to be significantly associated with hypertension, arthritis and functional disability [2]; hence, we treated them as confounding variables. For angina, the respondents were asked whether they had been diagnosed by a health professional with angina or angina pectoris (a heart disease). Respondents were also asked whether they had been diagnosed by a health professional with diabetes (high blood sugar). Both responses were scored as a binary variable (0) no or (1) yes.

#### Psychosocial factors

Depression and a person’s level of happiness have also been found to either increase or decrease the risk of hypertension and other illnesses in the youth and younger adults but rarely in the elderly [66]. Depression was developed as a construct out of four variables; have you ever been diagnosed with depression (0 = no, 1 = yes); during the last 12 months, have you had a period lasting several days when you felt sad, empty or depressed; have you had a period lasting several days when you lost interest in most things you usually enjoy such as personal relationships, work, or hobbies/recreation; and have you had a period lasting several days when you have been feeling your energy decreased or that you are tired all the time (0 = no, 1 = yes). The computation of these four variables produced a Cronbach’s alpha of 0.84 above the recommended threshold of 0.80 [67, 68]. A latent variable was created and labeled depression using principal components analysis. All four items loaded on the same latent construct with an eigenvalue of 2.67 explaining 66.9% of the variance in the items [1]. Level of happiness was measured as (1) happy, (2) neither happy nor unhappy, and (3) unhappy.

#### Behavioral/lifestyle factors

Healthy behavioral/lifestyle factors have been established as decreasing the risk of hypertension, arthritis, functional disability and promoting cognitive enhancement [69]. These factors include aerobic/physical activity, eating of vegetables and fruits, and reduction in alcohol consumption. In this study, we included a question on alcohol consumption, which was “have you ever consumed a drink that contains alcohol (such as beer, wine, spirits, etc.)?” coded as (0) no and (1) yes. We also added a question on engagement in physical activity. Respondents were asked “does your work involve vigorous-intensity activity that causes large increases in breathing or heart rate (like heavy lifting, digging, or chopping wood) for at least 10 minutes continuously?” This was coded as (0) no and (1) yes.

#### Socio-cultural and biosocial factors

Education, wealth, employment, religion, place of residence, sex and ethnicity provided socio-cultural and biosocial information on the participants. Respondents’ education was classified into (0) no education, (1) primary education, (2) secondary education, and (3) university education; a derived wealth variable was created from a series of questions that tapped into the wealth status of respondents. These questions were based on household ownership of durable goods, dwelling characteristics (type of floors, walls, etc.), and access to services (improved water, sanitation, and cooking fuel) for a total of 21 assets. A two-step random effect probit model was used to generate the wealth quintiles. We first created an asset ladder based on the endorsement rate of the different assets. This ladder was then used to arrange household assets on the same scale [70, 71]. This resulted in a continuous wealth score out of which the wealth quintiles were created and is here coded 0 = poorest, 1 = poorer, 2 = middle, 3 = richer, and 4 = richest); the employment status of participants coded 0 = not employed and 1 = employed; religious denomination coded 0 = none, 1 = Christian, 2 = Islam, 3 = Traditional, and 4 = Other; and place of residence coded 0 = urban and 1 = rural. Sex was coded as (1) male and (2) female, whereas respondents’ ethnicity was distinguished into five categories: (0) Akan, (1) Ewe, (2) Ga-Adangme, (3) Northern ethnic groups, and (4) Other ethnic groups

### Data analysis

First, we examined the univariate distribution of the outcome variables to determine the type of analysis to use. The categorical distribution of all six outcome variables allowed us to conduct Pearson chi-squared test associations between systolic and diastolic measures of hypertension, arthritis, difficulties with recall and learning new tasks, deficiencies in ADLs and IADLs and all the categorical independent variables. Since the omnibus Pearson chi-square value does not indicate which categories contribute to statistical significance for categorical variables with more than two categories, we used different logistic regression models at the bivariate level. This was then followed by multiple regression analysis, which included all variables that were significant at *p*<0.20.

We observed that the SAGE data had a hierarchical structure with participants nested within survey clusters [53], which could bias the standard errors. In order to adjust for dependence, we employed random effects models to estimate the magnitude and significance of clustering using Intra Class Correlation (ICC). ICC was calculated as the ratio of the variance at the cluster level to the sum of the variances at the individual and cluster levels [72, 73].

The outcome variables, systolic and diastolic hypertension had three unordered categories requiring the use of multinomial logistic regression. We identified normal/prehypertensive category as the base/reference group and estimated the probability of being stage 1 or stage 2- hypertension [74]. In order to adjust for clustering, we estimated a multilevel multinomial logit model using GLLAMM (generalized linear latent and mixed models) for unordered categorical responses (i.e. systolic and diastolic hypertension) [75]. We fitted generalized linear mixed effect models (GLME) with a binomial family distribution for binary outcome variables (arthritis and deficiencies in ADLs and IADLs) and an ordinal family distribution for ordinal outcome variables (difficulties with recall and learning a new task) with a logit link function [72, 73].

All analyses were performed using STATA version 14 (College Station, TX: StataCorp LP), variables considered significant at *p*-value ≤ 0.05.

### Ethical approval

The ethical responsibility for SAGE lies with the institutions that conducted the surveys in each country. WHO’s Ethical Review Board and the University of Ghana Medical School Ethics and Protocol Review Committee gave the ethical approval for SAGE Wave 1 in Ghana. Hence, we did not require a separate ethics approval for this study using the Ghana data [58, 76].

## Results

### Chronic diseases: Hypertension

Table 1 shows a significant relationship between the independent variables and the outcome variable–hypertension–except for angina and education. The systolic measures of hypertension showed a progression in the risk of stage 2 hypertension as respondents’ weight increase: underweight (14%), normal weight/prehypertension (20.72%), overweight (31.60%) and obese (67.14%). Similar for the diastolic measures of hypertension, the proportion of respondents grouped as having stage 2 hypertension increased significantly with increase in body weight: underweight (20.20%), normal weight (27.97%), overweight (36.11%) and obese (69.14%). In both systolic and diastolic measurements, a greater proportion of obese respondents were at risk of stage 2 hypertension.

**Table 1:** Univariate and bivariate results of elderly obesity and its association with hypertension.

	Systolic				Diastolic		
	N-size	NP^1^	St. 1^2^	St.2^3^	NP	St. 1	St.2
**Body Mass Index**							
Normal Weight (Ref.)	1,061	54.24	25.05	20.72	51.41	20.62	27.97
Underweight	391	65.73	20.20	14.07	61.38	18.41	20.20
Overweight	288	42.36	26.04	31.60	39.58	24.31	36.11
Obese	350	17.71	15.14	67.14***	17.17	13.14	69.14***
**Ever diagnosed with Angina (Ref. No)**	**1,832**	53.06	24.73	22.22	50.16	21.4	28.44
Yes	76	59.21	23.68	17.11	55.26	19.74	25.00
**Ever diagnosed with Diabetes (Ref. No)**	**1,836**	53.81	24.56	21.62	50.65	21.46	27.89
Yes	73	39.73	27.4	32.88*	42.47	17.81	39.73
**Depression**							
**Happiness (Ref.: Happy)**	**1,067**	53.61	25.02	21.37***	50.61	22.68	26.71
Neither	501	54.49	24.15	21.36	51.10	19.36	29.54
Unhappy	523	32.89	16.25	50.86***	31.74	13.00	55.26***
**Alcohol Consumption (Ref.:No)**	**855**	50.64	22.57	26.78	46.55	21.75	31.70
Yes	1,054	55.41	26.67	18.22***	53.42	20.97	25.62***
**Engaged in Vigorous Activity (No)**	**1,210**	53.97	24.38	21.65	52.31	21.57	26.12
Yes	699	52.07	25.18	22.75	46.92	20.89	32.19*
**Education (Ref.: None)**	**1,465**	48.26	21.77	29.97	45.39	18.63	35.97
Primary	328	53.05	22.26	24.70	47.87	21.65	30.47
Secondary	247	46.56	25.91	27.53	46.15	21.46	32.39
Tertiary	51	41.18	33.33	25.49	50.98	19.61	29.41
**Wealth Index (Ref.: Poorest)**	**450**	56.89	18.22	24.89	53.78	16.67	29.56
Poorer	444	49.32	25.45	25.23	44.59	21.89	33.56
Middle	441	46.26	23.13	30.61	43.31	18.14	38.55
Richer	399	44.36	23.81	31.83	41.60	20.45	37.84
Richest	357	45.10	22.69	32.21**	46.22	20.45	33.33***
**Religion (Ref.: None)**	**101**	50.50	26.76	22.77	49.5	12.87	37.62
Christianity	1,260	51.19	25.40	23.41	49.52	22.14	28.33
Islam	309	49.19	24.92	25.89	43.37	21.04	35.60
Traditional	225	70.22	19.11	10.67	63.56	21.33	15.11
Others	188	2.66	2.13	95.21***	4.26	0.00	95.74***
**Place of Residence (Ref.:Urban)**	**810**	42.59	24.32	33.09	41.11	20.49	38.40
Rural	1,281	52.46	21.55	26.00***	49.10	18.81	32.08***
**Sex (Ref.: Male)**	**987**	55.32	22.59	22.09	48.83	19.76	31.41
Female	1,104	42.66	22.64	34.69***	43.48	19.20	37.32*
**Ethnicity (Ref.: Akan)**	**914**	50.44	25.49	24.07	49.02	21.55	29.43
Ewe	136	52.21	27.21	20.59	52.94	22.06	25.00
Ga Adangme	190	55.26	22.11	22.63	50.53	20.00	29.47
Northern groups	197	51.27	26.40	22.34	46.19	24.37	29.44
Others	654	42.66	16.67	40.67***	38.99	14.37	46.64

Notes:

^1^NP = Normal + Prehypertension;

^2^St1 = stage 1 hypertension,

^3^St2 = stage 2 hypertension;

*p≤0.05,

**p≤0.01,

***p≤0.001

The proportion of respondents diagnosed with diabetes at risk of stage 2 hypertension (32.88%) was significantly greater for systolic measures. The risk of stage 2 hypertension was significantly greater for respondents grouped as unhappy (50.86%), richest wealth quintile (32.21%), practice other religions (95.21%), females (34.69%) and other ethnic groups (40.67%). Similar results were found for diastolic measures of hypertension. Also, the proportion of respondents at risk of stage 1 hypertension were significantly greater for respondents who reported alcohol consumption (26.67%), were in the richest wealth quintile (22.69%), and were of the Ewe ethnic group (27.21%) (Table 1).

After adjusting for all variables significant at the bivariate level (*p*<0.2) for systolic measures of hypertension, the expected risk of stage 1 hypertension instead of normal/prehypertension decreased by 35% for respondents who were underweight and increased by 64% for obese elderly relative to those with normal weight (Table 2). Also, the expected risk of stage 2 hypertension instead of normal/prehypertension decreased by 43% for respondents who were underweight and increased by 73% and 81% for the overweight and obese, respectively. For diastolic measures of hypertension, the expected risk of stage 1 hypertension instead of normal/prehypertension increased by 51% for overweight and 79% for the obese after adjusting for all variables. Similarly, the expected risk of stage 2 hypertension instead of normal /prehypertension, increased by 67% and 77% for overweight and obese relative to normal weight. Further, our sub-analysis using waist circumference showed that women with greater abdominal fat mass (WC>88cm) had an increased risk of stage 2 hypertension (aRRR = 2.02, *p*<0.001, 95% CI: 1.40–2.90) than normal/prehypertension. Men with greater abdominal fat mass (>102cm) did not show significant associations with hypertension. Together, these results fail to reject the hypothesis that elderly obesity increase the expected risk of stage 1 and stage 2 hypertension.

**Table 2:** Multilevel multinomial logit model of elderly obesity and its effect on stage 1 and stage 2 hypertension using systolic and diastolic unordered measures.

	Systolic (aRRR^1^)		Diastolic (aRRR)	
	Stage 1 HTN^2^	Stage 2 HTN	Stage 1 HTN	Stage 2 HTN
**Body Mass Index**				
Normal Weight (Ref^3^.)	1.00	1.00	1.00	1.00
Underweight	0.65(0.40, 0.81) **	0.57 (0.41, 0.81) ***	0.75 (0.54, 1.02)	0.63 (0.47,0.85)
Overweight	1.25(0.89, 1.77)	1.73 (1.24, 2.40) ***	1.51 (1.06, 2.15) *	1.67 (1.21, 2.31) ***
Obese	1.64(1.07, 2.51) *	1.81 (1.19, 2.77) **	1.79 (1.16, 2.77) **	1.77 (1.18, 2.63) **
**Depression**	1.04(0.98, 1.11)	0.98(0.91, 1.05)	1.06 (0.99, 1.13)	1.01 (0.95, 1.07)
**Happiness (Ref.: Happy)**	1.00	1.00	1.00	1.00
Neither	1.01 (0.76, 1.32)	1.08 (0.80, 1.44)	0.89 (0.67, 1.19)	1.16 (0.89, 1.51)
Unhappy	0.98 (0.71, 1.36)	1.17 (0.85, 1.63)	0.88 (0.63, 1.24)	1.19 (0.89, 1.61)
**Alcohol Consumption**	1.00	1.00	1.00	1.00
Yes	1.41(1.08, 1.85) *	0.89 (0.68, 1.18)	0.94 (0.71, 1.23)	0.91 (0.71, 1.16)
**Wealth Index (Ref.: Poorest)**	1.00	1.00	1.00	1.00
Poorer	1.47 (1.02, 2.09) *	1.03 (0.70, 1.53)	1.47 (1.03, 2.13) *	1.32 (0.94, 1.86)
Middle	1.38 (0.95, 1.99)	1.33 (0.91, 1.95)	1.19 (0.81, 1.74)	1.45 (1.02, 2.03)
Richer	1.40 (0.95, 2.05)	1.36 (0.91, 2.03)	1.26 (0.85, 1.88)	1.34 (0.93, 1.93)
Richest	1.17 (0.76, 1.79)	1.10 (0.71, 1.69)	1.06 (0.66, 1.54)	0.82 (0.54, 1.23)
**Religion (Ref.: None)**	1.00	1.00	1.00	1.00
Christianity	0.87(0.52, 1.46)	0.73 (0.43, 1.26)	1.56 (0.83, 2.92)	0.66 (0.41, 1.05)
Islam	1.11 (0.60, 2.05)	1.01 (0.53, 1.91)	1.69 (0.81, 3.51)	1.00 (0.57, 1.73)
Traditional	0.58 (0.31, 1.07)	0.38 (0.19, 0.75) **	1.45 (0.72, 2.93)	0.35 (0.19, 0.64***
Others	1.19 (0.24, 5.76)	1.15 (0.25, 5.27)	0.30(0.11, 0.82) **	0.63 (0.15, 2.16)
**Place of Residence (Ref.:Urban)**	1.00	1.00	1.00	1.00
Rural	0.86 (0.65, 1.13)	0.79 (0.59, 1.05)	0.91 (0.69, 1.19)	0.79 (0.61, 1.02)
**Sex (Ref.: Male)**	1.00	1.00	1.00	1.00
Female	1.33 (1.04, 1.69) *	1.66 (1.28, 2.15) ***	0.91 (0.69, 1.19)	0.79 (0.61, 1.01)
**Ethnicity (Ref.: Akan)**	1.00	1.00	1.00	1.00
Ewe	1.05 (0.66, 1.67)	0.86 (0.52, 1.43)	1.01 (0.63, 1.62)	0.86 (0.54, 1.37)
Ga Adangme	0.76 (0.50, 1.16)	0.88 (0.57, 1.36)	0.93 (0.60, 1.44)	1.03 (0.71, 1.53)
Northern groups	0.99 (0.65, 1.51)	0.82 (0.52, 1.29)	1.16 (0.75,1.79)	0.91 (0.61, 1.34)
Others	0.93 (0.64, 1.34)	0.83 (0.55, 1.23)	0.93 (0.62, 1.38)	0.90 (0.64, 1.27)

Notes:

^1^aRRR = Adjusted Relative Risk Ratios;

^2^HTN = Hypertension;

^3^Reference categories;

*p≤0.05,

**p≤0.01,

***p≤0.001

### Chronic diseases: Arthritis

From Table 3, the proportion of respondents who had been diagnosed with arthritis was significantly greater for obese elderly respondents (24.71%) than for normal weight (15.27%). Additionally, the proportion of participants diagnosed with arthritis was significantly greater for those diagnosed with angina (31.58%), engaged in vigorous activity (20.89%), and females (20.35%), but lower for respondents in the other ethnic cohort (11.37%), males (14.18%) and the Akan ethnic group (20.50%).

**Table 3:** Univariate, bivariate and adjusted results of generalized linear mixed effect logit model of arthritis on elderly obesity.

	N-size	Univariate		Bivariate	Adjusted model	
		No	Yes	OR (95%CI)	aOR	(95% CI)
**Body Mass Index**						
Normal Weight (Ref.)	1,061	84.73	15.27	1.00	1.00	
Underweight	391	81.33	18.67	1.28 (0.89, 1.83)	1.08	(0.74, 1.58)
Overweight	287	81.18	18.67	1.36 (0.93, 2.02)	1.20	(0.79, 1.83)
Obese	170	75.29	24.71*	1.94 (1.22, 3.06) **	1.70	(1.04, 2.78) *
**Ever diagnosed with Angina (Ref. No)**	**1,525**	83.24	16.76	1.00	1.00	
Yes	76	68.42	31.58***	2.07 (1.11,3.83) *	2.22	(1.25, 4.52) **
**Ever diagnosed with Diabetes (Ref. No)**	**1,836**	82.41	1759	1.00		
Yes	73	89.04	10.96	0.63 (0.27, 1.41)		
**Depression**				1.36 (1.27, 1.45)***	1.35	(1.26, 1.46) **
**Happiness (Ref.: Happy)**	**1,067**	82.66	17.34	1.00		
Neither	501	83.83	16.17	0.87 (0.62, 1.22)		
Unhappy	341	80.94	19.06	1.18 (0.82, 1.70)		
**Alcohol Consumption (Ref.:No)**	**855**	81.4	18.60	1.00		
Yes	1,054	83.68	16.32	0.89 (0.67, 1.17)		
**Engaged in Vigorous Activity (No)**	**1,210**	84.71	15.29	1.00	1.00	
Yes	699	79.11	20.89**	1.49 (1.12, 1.97) **	1.24	(0.67, 2.26)
**Education (Ref.: None)**	**1,312**	82.47	17.53	1.00		
Primary	314	83.44	16.56	089 (0.61, 1.33)		
Secondary	237	81.86	18.14	1.13 (0.75, 1.72)		
Tertiary	46	86.96	13.04	0.91 (0.30, 2.75)		
**Wealth Index (Ref.: Poorest)**	**413**	85.96	14.04	1.00	1.00	
Poorer	406	79.8	20.2	1.54 (1.01, 2.37) *	1.24	(0.79, 1.95)
Middle	402	84.83	15.17	1.11 (0.72, 1.72)	0.86	(0.54,1.39)
Richer	366	80.05	19.95	1.34 (0.87, 2.03)	1.32	(0.82, 2.14)
Richest	322	82.30	17.70	1.29 (0.82, 2.05)	1.17	(0.69, 1.98)
**Religion (Ref.: None)**	**100**	85.00	15.00	1.00		
Christianity	1,259	81.25	18.75	1.32 (0.64, 2.72)		
Islam	306	83.66	16.34	1.26 (0.57, 2.76)		
Traditional	224	88.39	11.61	0.85 (0.36, 1.98)		
Others	12	75	25	2.49 (0.51, 12.13)		
**Place of Residence (Ref.: Urban)**	**739**	83.09	16.91	1.00		
Rural	1,170	82.39	17.61	1.06 (0.79, 1.41)		
**Sex (Ref.: Male)**	**931**	85.82	14.18	1.00	1.00	
Female	978	79.65	20.35***	1.44 (1.08, 1.91) *	1.43*	(0.06, 1.94)
**Ethnicity (Ref.: Akan)**	**912**	79.5	20.50	1.00	1.00	
Ewe	136	86.03	13.97	0.55 (0.31, 0.94) *	0.83	(0.43, 1.62)
Ga Adangme	190	82.63	17.37	0.81 (0.49, 1.30)	0.94	(0.53, 1.64)
Northern groups	196	80.61	19.39	0.86 (0.56, 1.35)	0.90	(0.53, 1.54)
Others	475	88.63	11.37***	0.48 (0.34, 0.71)***	0.49	(0.30, 0.80) **
**Log Likelihood**					-758.40	
**Significance (Wald** χ^2^)					102.77 (15) ***	
**ICC**					0.32(0.23, 0.41) ***	

**Notes:** OR = Odds Ratios; aOR = adjusted Odds Ratios; ICC = Intra Class Correlation; CI: Confidence Intervals;

*p≤0.05,

**p≤0.01,

***p≤0.001

After adjusting for significant factors at the bivariate level (*p*<0.2), the odds of obese elderly diagnosed with arthritis increased by a factor of 70%. Those who had been diagnosed with angina were 122% more likely to be diagnosed with arthritis. Also, respondents who reported depression and females were 35% and 43% more likely to be diagnosed with arthritis, respectively. The univariate, bivariate and adjusted results fail to reject the second hypothesis that obese elderly respondents are more likely to be diagnosed with arthritis than those with normal weight.

### Cognitive impairment: Difficulties with recall

Of the 288 and 174 participants who were grouped as overweight and obese respectively, 5.56% and 9.20% reported experiencing severe difficulties with recall (Table 4). The proportion of respondents who reported severe difficulties with recall were significantly greater for those diagnosed with angina (18.42%), who reported being unhappy (20.75%), and females (14.01%). However, the proportion of respondents who reported severe difficulties with recall was significantly lower for those who engaged in vigorous activity (7.73%).

**Table 4:** Univariate, bivariate and adjusted results of generalized linear mixed effect ordinal logit models of difficulties with recall on elderly obesity.

	N-size	Univariate				Bivariate	Adjusted model	
		None	Mild	Mod.	Severe	OR^1^ (95% CI^2^)	aOR^3^	(95% CI)
**Body Mass Index**								
Normal Weight (Ref.)	1,061	25.45	34.87	28.46	11.22	1.00	1.00	
Underweight	391	25.58	28.13	34.27	12.02	1.14 (0.89, 1.46)	1.05	(0.84, 1.33)
Overweight	288	30.90	37.85	35.69	5.56	0.69 (0.54, 0.90) **	0.71	(0.54, 0.92) *
Obese	174	31.61	29.31	29.89	9.20**	0.69 (0.47, 1.01)	0.79	(0.57, 1.12)
**Ever diagnosed with Angina (Ref. No)**	**1,831**	26.76	34.13	29.06	10.05	1.00		
Yes	76	26.32	18.42	36.84	18.42**	1.39 (0.74, 2.62)		
**Ever diagnosed with Diabetes (Ref. No)**	**1,835**	26.98	33.57	28.99	10.46	1.00		
Yes	73	20.55	31.51	39.73	8.22	1.19 (0.80, 1.78)		
**Depression**						1.07 (1.01, 1.13) *	1.02	(0.97, 1.08)
**Happiness (Ref.: Happy)**	**1,066**	31.89	37.00	22.61	7.89	1.00	1.00	
Neither	501	22.55	29.14	39.52	8.78	1.72 (1.35, 2.17)***	1.67	(1.34, 2.06) ***
Unhappy	347	17.58	26.22	35.45	20.75***	3.03 (2.27, 4.04)***	2.61	(2.02, 3.38) ***
**Alcohol Consumption (Ref. No)**	**855**	24.68	34.39	29.47	11.46	1.00		
Yes	1,053	28.40	32.76	29.34	9.50	0.84 (0.69, 1.02)		
**Engaged in Vigorous Activity**	**1,209**	25.14	29.78	33.17	11.91	1.00	1.00	
Yes	699	29.47	39.91	22.89	7.73***	0.68 (0.55, 0.82)***	0.57	(0.46, 0.71) ***
**Education (Ref.: None)**	**1,316**	23.02	32.67	32.60	11.70	1.00	1.00	
Primary	314	29.62	35.99	26.11	8.28	0.67 (0.51, 0.88) **	0.68	(0.53, 0.88) **
Secondary	237	43.46	31.65	18.57	6.33	0.44 (0.31, 0.61)***	0.50	(0.36, 0.68) ***
Tertiary	47	31.91	46.81	14.89	6.38***	0.53 (0.25, 1.09)	0.65	(0.36, 1.17)
**Wealth Index (Ref.: Poorest)**	413	22.03	32.69	33.17	12.11	1.00	1.00	
Poorer	408	26.72	32.6	29.17	11.52	0.77 (0.57, 1.01)	0.95	(0.72, 1.24)
Middle	402	25.37	34.33	29.60	10.70	0.87 (0.67, 1.13)	0.92	(0.70, 1.21)
Richer	367	27.79	35.69	28.34	8.17	0.65 (0.48, 0.87)**	0.99	(0.73, 1.33)
Richest	324	33.95	31.79	25.62	8.64	0.55 (0.39, 0.74)***	0.98	(0.70, 1.38)
**Religion (Ref.: None)**	**101**	18.81	31.68	33.66	15.84	1.00		
Christianity	1,259	26.29	34.47	28.36	10.88	0.65 (0.41, 1.04)		
Islam	309	26.86	32.36	29.77	11.00	0.68 (0.41, 1.12)		
Traditional	225	33.78	29.78	32.00	4.44	0.52 (0.31, 0.88) *		
Others	12	25.00	33.33	33.33	8.33	0.49 (0.16, 1.57)		
**Place of Residence (Ref.: Urban)**	741	29.55	32.52	27.94	9.99	1.00	1.00	
Rural	1,173	25.15	34.02	30.26	10.57	1.27 (1.04, 1.57) *	1.29	(0.95, 1.75)
**Sex (Ref.: Male)**	**936**	32.91	34.83	25.75	6.52	1.00	1.00	
Female	978	21.06	32.11	32.82	14.01***	1.92(1.57, 2.33)***	1.54	(1.27, 1.87) ***
**Ethnicity (Ref.: Akan)**	**914**	27.35	34.79	26.04	11.82	1.00		
Ewe	136	24.26	30.15	36.76	8.82	1.40 (0.98, 2.00)		
Ga Adangme	190	31.05	25.26	33.68	10.00	1.11 (0.76, 1.61)		
Northern groups	197	23.35	34.52	35.03	7.11	1.15 (0.87, 1.52)		
Others	477	26.42	34.59	29.56	9.43	1.12 (0.89, 1.42)		
**Log Likelihood**							-2353.29	
**Significance (Wald** χ^2^)							191.66 (16) ***	
**Level 2 Variance**							0.67 (0.48, 0.96) ***	

Notes:

^1^OR = Odds Ratios;

^2^CI: Confidence Intervals;

^3^aOR = Odds Ratios; Mod.: Moderate;

*p≤0.05,

**p≤0.01,

***p≤0.001; Ref: Reference category

After adjusting for the effect of all factors significant at the bivariate level (*p*<0.2), the likelihood of overweight respondents reporting severe difficulties with recall decreased by 29% relative to those with normal weight (Table 4). Similarly, those engaged in vigorous activity and those with secondary education were 43% and 50% less likely to report severe difficulties with recall. The unhappy respondents were 161% more likely to report difficulties with recall compared to the happy respondents. Also, females were 54% more likely to report severe difficulties with recall relative to males. The hypothesis of obese elderly respondents reporting severe difficulties with recall was rejected by the results.

### Cognitive impairment: Difficulties with learning a new task

The proportion of respondents who reported severe difficulties with learning new tasks was significantly greater for obese elderly (12.07%) than those with normal weight (Table 5). Respondents diagnosed with angina who reported difficulties with learning new tasks were proportionally greater (19.74%). Further, the proportion of respondents reporting severe difficulties with learning new task was greater for unhappy (21.61%) and female (14.11%) respondents. The proportion of respondents who reported difficulties with learning new tasks was lower for those who consumed alcohol (9.12%) and those with tertiary education (6.38%).

**Table 5:** Univariate, bivariate and adjusted results of generalized linear mixed effect ordinal logit models of difficulties with learning a new task on elderly obesity.

	N-size	Univariate				Bivariate	Adjusted model	
		None	Mild	Mod.	Severe	OR^1^ (95%CI^2^)	aOR^3^	(95% CI)
**Body Mass Index**								
Normal Weight (Ref.)	1,061	28.09	32.14	30.35	9.43	1.00	1.00	
Underweight	391	26.34	27.62	31.20	14.83	1.20 (0.93,1.54)	1.17	(0.93, 1.47)
Overweight	288	34.38	31.94	25.69	7.99	0.75 (0.57, 0.98) *	0.75	(0.57, 0.97) *
Obese	174	30.46	27.01	30.46	12.07*	0.85 (0.59, 1.22)	0.96	(0.70, 1.35)
**Ever diagnosed with Angina (Ref. No)**	**1,831**	29.00	31.08	29.71	10.21	1.00		
Yes	76	23.68	23.68	32.89	19.74*	1.60 (0.85, 2.99)		
**Ever diagnosed with Diabetes (Ref. No)**	**1,835**	28.88	30.95	29.75	10.41	1.00		
Yes	73	26.03	26.03	32.88	15.07	1.19 (0.74, 1.87)		
**Depression**						1.10 (1.03, 1.17) **	1.06	(1.01, 1.11) *
**Happiness (Ref.: Happy)**	**1,066**	35.27	34.33	24.11	6.29	1.00	1.00	
Neither	501	21.56	29.34	37.13	11.98	1.91 (1.50, 2.42)***	1.95	(1.57, 2.41) ***
Unhappy	347	19.88	21.61	36.89	21.61***	3.15 (2.32, 4.27)***	2.80	(2.16, 3.63) ***
**Alcohol Consumption (Ref.:No)**	**855**	26.78	29.82	30.99	12.40	1.00		
Yes	1,053	30.39	31.53	28.96	9.12*	0.79 (0.64, 0.95)		
**Vigorous Activity (No)**	**1,209**	27.46	28.7	30.69	13.15	1.00	1.00	
Yes	699	31.04	34.33	28.47	6.15***	0.74 (0.61, 0.90) *	0.66	(0.53, 0.81) *
**Education (Ref.: None)**	**1,316**	23.94	31.91	31.61	12.54	1.00	1.00	
Primary	314	32.48	28.98	29.94	8.60	0.69 (0.51, 0.91) **	0.67	(0.52, 0.87) **
Secondary	237	47.68	27.85	21.52	2.95	0.40 (0.29, 0.54)***	0.39	(0.28, 0.53) ***
Tertiary	47	48.94	23.40	21.28	6.38***	0.47 (0.19, 1.13)	0.50	(0.27, 0.93) *
**Wealth Index (Ref.: Poorest)**	**413**	23.49	32.69	32.45	11.38	1.00		
Poorer	408	27.94	31.62	29.41	11.03	0.73 (0.44, 1.22)		
Middle	402	27.36	28.36	33.08	11.19	0.72 (0.42, 1.25)		
Richer	367	31.34	31.34	28.69	8.89	0.66 (0.38, 1.15)		
Richest	324	36.11	29.32	24.69	9.88	0.35 (0.11, 1.14)		
**Religion (Ref.: None)**	**101**	24.75	27.72	28.71	18.81	1.00		
Christianity	1,259	28.51	30.82	29.31	11.36	0.74 (0.44, 1.22)		
Islam	309	29.13	33.01	29.45	8.41	0.72 (0.41, 1.22)		
Traditional	225	32.44	27.56	35.11	4.89	0.66 (0.38, 1.15)		
Others	12	33.33	33.33	8.33	25.00*	0.35 (0.11, 1.14)		
**Place of Residence (Ref.: Urban)**	**741**	30.09	29.96	29.28	10.66	1.00		
Rural	1,173	28.13	31.2	30.18	10.49	1.15 (0.93, 1.40)		
**Sex (Ref.: Male)**	**936**	36.22	30.98	25.96	6.84	1.00	1.00	
Female	978	21.88	30.47	33.54	14.11***	1.99 (1.64, 2.42)***	1.49	(1.22, 1.81) ***
**Ethnicity (Ref.: Akan)**	**914**	29.32	30.09	28.23	12.36	1.00	1.00	
Ewe	136	30.88	25.00	36.03	8.09	1.10 (0.75, 1.60)	0.89	(0.65, 1.49)
Ga Adangme	190	33.16	21.05	34.21	11.58	1.11 (0.75, 1.63)	1.03	(0.71, 1.49)
Northern groups	197	24.87	39.59	27.41	8.12	1.07 (0.80, 1.42)	0.98	(0.70, 1.37)
Others	477	27.46	33.75	30.4	8.39**	1.10 (0.87, 1.39)	1.05	(0.78, 1.40)
Log Likelihood							-2356.51	
Significance (Wald χ^2^)							221.20 (15) ***	
**Level 2 Variance**							0.62 (0.43, 0.89) ***	

Notes:

^**1**^OR = Odds Ratios;

^2^CI: Confidence Intervals;

^**3**^OR = Adjusted Odds Ratios; Mod: Moderate;

*p≤0.05,

**p≤0.01,

***p≤0.001

After adjusting for the effect of all factors significant at the bivariate level (*p*<0.2), participants who were overweight were 25% less likely to have severe difficulties with learning a new task. Participants who reported being unhappy were 180% more likely to report severe difficulties with learning new tasks. Also, females compared to males were 49% more likely to report severe difficulties with learning new tasks. Respondents who engaged in vigorous activities and those with tertiary education were 34% and 50% less likely to report severe difficulties with learning new tasks. Based on these results, our hypothesis that elderly obese respondents would report severe difficulties with learning new tasks was rejected.

### Functional disability: Deficiencies with activities of daily living (ADLs)

The proportion of respondents who reported severe deficiencies in ADLs was significantly greater for obese elderly respondents (68.67%) than those with normal weight (55.11%) (Table 6). Similar trends were identified with respondents diagnosed with angina (83.56%), those who reported unhappy (77.81%) and females (54.44%). The proportion of respondents who reported deficiencies in ADLs was significantly lower for those who reported alcohol consumption (55.70%), engaged in vigorous activities (45.10%), and those with tertiary education (40%).

**Table 6:** Univariate, bivariate and adjusted results of generalized linear mixed effect logit models of activities of daily living (ADLs) on elderly obesity.

	N-size	Univariate		Bivariate	Adjusted model
		No Def^1^.	Def^2^.	OR^3^ (95%CI^4^)	OR (95%CI)
**Body Mass Index**					
Normal Weight (**Ref**.)	998	44.89	55.11	1.00	1.00
Underweight	363	33.61	66.39	1.64(1.22, 2.19) ***	1.71 (1.26, 2.34) **
Overweight	263	38.78	61.22	1.16(0.84, 1.59)	1.21 (0.85, 1.72)
Obese	150	31.33	68.67***	1.48 (0.96, 2.26)	1.59 (0.99, 2.55)
**Ever diagnosed with Angina (Ref. No)**	1,694	41.5	58.5	1.00	1.00
Yes	73	16.44	83.56***	4.16(1.93, 8.96) ***	3.12 (1.78, 10.42) ***
**Ever diagnosed with Diabetes (Ref. No)**	1,704	40.55	59.45	1.00	
Yes	64	37.5	62.5	1.16 (0.66, 2.05)	
**Depression**				1.06 (0.99, 1.34)	
**Happiness (Ref.: Happy)**	989	47.83	52.17	1.00	1.00
Neither	474	37.34	62.66	1.49 (1.14, 1.97) **	1.55 (1.17, 2.06) *
Unhappy	311	22.19	77.81***	3.37 (2.43, 4.68) ***	2.69 (1.86, 3.89) ***
**Alcohol Consumption**	795	35.72	64.28	1.00	1.00
Yes	973	44.3	55.70***	0.79 (0.63, 0.99) *	0.91 (0.68, 1.20)
**Vigorous Activity**	1,085	31.34	68.66	1.00	1.00
Yes	683	54.9	45.10***	0.44 (0.34, 0.55) ***	0.34 (0.25, 0.45) ***
**Education (Ref.: None)**	1,217	36.57	63.43	1.00	1.00
Primary	293	44.03	55.97	0.69 (0.51, 0.96) *	0.61 (0.43, 0.85) **
Secondary	219	53.88	46.12	0.54 (0.39, 0.76) ***	0.51 (0.34, 0.77) ***
Tertiary	45	60	40.00***	0.48 (0.22, 1.02)	0.39 (0.18, 0.84) **
**Wealth Index (Ref.: Poorest)**	385	37.4	62.6	1.00	1.00
Poorer	376	42.82	57.18	0.68 (0.49, 0.95) *	0.81 (0.56, 1.17)
Middle	371	39.62	60.38	0.84 (0.59, 1.17)	0.88 (0.60, 1.28)
Richer	343	39.65	60.35	0.77 (0.54, 1.09)	0.95 (0.63, 1.42)
Richest	299	43.81	56.19	0.64 (0.45, 0.91) *	0.92 (0.59, 1.43)
**Religion (Ref.: None)**	88	36.36	63.64	1.00	1.00
Christianity	1,168	36.9	63.1	0.84 (0.50, 1.41)	1.00 (0.56, 1.77)
Islam	284	48.24	51.76	0.54 (0.31, 0.95) *	0.64 (0.33, 1.26)
Traditional	215	51.63	48.37	0.66 (0.37, 1.19)	0.68 (0.35, 1.32)
Others	11	54.55	45.45***	0.49 (0.12, 1.99)	0.35 (0.07, 1.83)
**Place of Residence (Ref.:Urban)**	675	39.56	60.44	1.00	
Rural	1,099	41.13	58.87	1.07 (0.856, 1.36)	
**Sex (Ref.: Male)**	887	52.54	47.46	1.00	1.00
Female	887	28.52	71.48***	2.35 (1.86, 2.96) ***	2.04 (1.56, 2.67) ***
**Ethnicity (Ref.: Akan)**	854	37.35	62.65	1.00	1.00
Ewe	120	35	65	1.08 (0.69, 1.69)	1.01 (0.58, 1.75)
Ga Adangme	167	33.53	66.47	1.23 (0.82, 1.83)	1.08 (0.66, 1.77)
Northern groups	183	53.01	46.99	0.59 (0.41, 0.84) **	0.59 (0.37, 0.94) *
Others	450	45.56	54.44***	0.89 (0.67, 1.17)	1.02 (0.65, 1.57)
Log Likelihood					-1003.77
Sig. (Wald χ^2^)					206.75(24) ***
**ICC**					0.22 (0.16,0.30) ***

Notes:

^1^No Def = No deficiencies in ADLs;

^2^Def = Severe functional Deficiencies;

^3^OR = Odds Ratios;

^4^CI: Confidence Intervals; ICC: Intraclass Correlation;

*p≤0.05,

**p≤0.01,

***p≤0.001

After adjusting for all factors significant at the bivariate level (*p*<0.2), respondents who grouped as underweight were 71% more likely to report severe functional deficiencies in ADLs (Table 6). Those diagnosed with angina were 212% more likely to report severe deficiencies in ADLs relative to those who were not. Respondents who reported unhappiness were also 169% more likely to report severe deficiencies with ADLs. Most significantly, those who engaged in vigorous activities were 66% less likely to report severe deficiencies with ADLs relative to those who did not. Likewise, those with secondary and tertiary education were 49% and 61% less likely to report severe deficiencies with ADLs compared to those with no education, respectively. Females were 104% more likely to report severe deficiencies with ADLs than males. These results reject our hypothesis that elderly obese respondents will report severe deficiencies with ADLs deficiencies.

### Functional disability: Deficiencies with instrumental activities of daily living

Table 7 with a focus on IADLs showed respondents who were obese had significantly severe deficiencies with IADLs (38.36%) than those who were categorized as normal weight (21.55%). The proportion of respondents diagnosed with angina (45.83%) and diabetes (44.93%) had severe deficiencies with IADLs. Proportionally, respondents who reported ever consuming alcohol (23.86%) had more severe deficiencies than those who did not. The proportion of respondents reporting severe deficiencies with IADLs was significantly greater for females (34.44%) than for males (16.96%).

**Table 7:** Univariate, bivariate and adjusted results of generalized linear mixed effect logit model of instrumental activities of daily living (IADLs) on elderly obesity.

	N-size	Univariate		Bivariate	Adjusted model
		No Def.	Def^1^.	OR^2^ (95%CI^3^)	aOR^4^ (95%CI)
**Body Mass Index**					
Normal Weight (Ref.)	1,030	78.45	21.55	1.00	1.00
Underweight	378	69.84	30.16	1.65 (1.21,2.25) **	1.59 (1.16, 2.18) **
Overweight	279	71.68	28.32	1.52 (1.07,2.15) *	1.47 (1.03, 2.11) *
Obese	159	61.64	38.36***	1.84 (1.23, 2.78) **	2.20 (1.42, 3.43) **
**Ever diagnosed with Angina**	1,767	74.99	25.01	1.00	1.00
Yes	72	54.17	45.83***	3.11(1.73, 5.59) ***	2.07 (1.17, 3.67) **
**Ever diagnosed with Diabetes**	1,771	74.87	25.13	1.00	1.00
Yes	69	55.07	44.93***	2.07 (1.21,3.57) **	2.45 (1.33, 4.50) *
**Depression**				1.04 (0.97, 1.12)	
**Happiness (Ref.: Happy)**	1,027	80.82	19.18	1.00	1.00
Neither	489	73.62	26.38	1.64 (1.21, 2.23) ***	1.73 (1.28, 2.34) **
Unhappy	330	54.55	45.45***	3.44 (2.53, 4.68) ***	4.25 (3.04, 5.94) ***
**Alcohol Consumption**	830	71.69	28.31	1.00	
Yes	1,010	76.14	23.86*	0.91 (0.71, 1.16)	
**Vigorous Activity**	1,150	72	28	1.00	
Yes	690	77.68	22.32**	0.79 (0.61, 1.02)	
**Education (Ref.: None)**	1,264	71.36	28.64	1.00	1.00
Primary	306	77.45	22.55	0.72 (0.51, 1.03)	0.73 (0.51, 1.04)
Secondary	230	83.48	16.52	0.52 (0.34, 0.81) **	0.61 (0.38, 0.98) *
Tertiary	46	84.78	15.22***	0.56 (0.19, 1.57)	0.55 (0.22, 1.39)
**Wealth Index (Ref.: Poorest)**	395	74.68	25.32	1.00	
Poorer	393	73.79	26.21	0.99 (0.68, 1.45)	
Middle	389	72.49	27.51	1.14 (0.78, 1.65)	
Richer	356	75	25	0.94 (0.64, 1.39)	
Richest	313	75.4	24.6	0.78 (0.52, 1.17)	
**Religion (Ref.: None)**	95	66.32	33.68	1.00	1.00
Christianity	1,214	71.91	28.09	0.63 (0.37, 1.06)	0.71 (0.41, 1.25)
Islam	299	79.26	20.74	0.39 (0.22, 0.72) **	0.51 (0.26, 0.99) *
Traditional	218	83.49	16.51	0.43 (0.22, 0.82) *	0.44 (0.22, 0.89) *
Others	12	58.33	41.67***	1.07 (0.28, 4.13)	1.30 (0.29, 5.88)
**Place of Residence (Ref.:Urban)**	710	72.39	27.61	1.00	1.00
Rural	1,136	75.35	24.65	0.95 (0.74, 1.22)	0.92 (0.65, 1.32)
**Sex (Ref.: Male)**	914	83.04	16.96	1.00	1.00
Female	932	65.56	34.44***	2.28 (1.76, 2.96) ***	2.22 (1.68, 2.94) ***
**Ethnicity (Ref.: Akan)**	894	70.25	29.25	1.00	1.00
Ewe	127	74.8	25.2	0.78 (0.48, 1.27)	0.89 (0.51, 1.55)
Ga Adangme	175	77.71	22.29	0.74 (0.47, 1.19)	0.64 (0.39, 1.05)
Northern groups	191	83.77	16.23	0.53 (0.32, 0.85) **	0.49 (0.26, 0.73) **
Others	459	76.47	23.53	0.77 (0.56, 1.04)	1.18 (0.75, 1.83)
**Log Likelihood**					914.04
**Sig. (Wald χ2)**					173.09(21) ***
**ICC**^**5**^					0.18 (0.12, 0.26) ***

Notes:

^1^Def.: Severe functional deficiencies;

^2^OR = Odds Ratios;

^3^CI: Confidence Intervals;

^4^aOR = Adjusted Odds Ratios; ICC: Intraclass correlation; Sig.: Model significance;

*p≤0.05,

**p≤0.01,

***p≤0.001

Adjusting for all factors significant at the bivariate level (*p*<0.20), respondents who were underweight were 59% more likely to report severe IADL deficiencies. Those who were overweight and obese were 47% and 120% more likely to report severe IADLs deficiencies than those grouped as normal weight. Angina and diabetes diagnosis increased the likelihood of reporting deficiencies in IADLs by 107% and 145%, respectively. Respondents who reported being unhappy were 325% more likely to report severe IADLs deficiencies. Next, those with secondary education were 39% less likely to report severe IADLs deficiencies. Interestingly, respondents who reported practicing Islam (49%) and traditional religion (56%) were less likely to report severe IADLs deficiencies relative to those who practiced no religion. Females were 122% more likely to report severe deficiencies with IADLs. These results fail to reject the hypothesis that obese elderly respondents would report more severe deficiencies with IADLs than those with normal weight.

## Discussion

This study examined the health risks associated with obesity among the elderly in Ghana. Specifically, we investigated whether elderly obesity is associated with higher risks of hypertension, arthritis, difficulties with recall and learning new task, and severe deficiencies with ADLs and IADLs. We found an increased risk of both stage 1 and stage 2 hypertension for overweight and obese respondents and a significantly lower risk for underweight respondents in both systolic and diastolic measures. Adjusting appropriately for body fat among the elderly, our use of waist circumference showed that women with visceral obesity had a significantly higher risk of stage 2 hypertension.

These findings suggest a progressive increase in the potential risk of cardiovascular diseases if the elderly overweight receive no immediate intervention. Lifestyle interventions including regular physical activity, consumption of fruits, vegetables and low-fat dairy products, lower dietary sodium intake, and moderation of alcohol use have been shown to be effective for individuals diagnosed with stage 1 hypertension [69]. However, individuals whose blood pressure suggests they may be suffering from stage 2 hypertension require prompt medical treatment in addition to lifestyle modifications [1, 77]. Delayed interventions could lead to rapid progression from stage 1 to stage 2 hypertension, which has more severe health complications.

Rising levels of cardiovascular diseases in Ghana is blamed on a lack of appropriate interventions and inadequate allocation of resources toward health education programs [57]. Our study findings call for primary prevention programs geared towards mass screening, treatment of high risk individuals, and regular assessments of potential cardiovascular risks among the elderly, a view also shared by other scholars [78]. Further, a more expansive health education campaign aimed at lifestyle and dietary changes should be directed to the public to increase awareness of the different stages of hypertension.

The finding that the obese elderly population were 70% more likely to be diagnosed with arthritis has important implications. While this finding confirms previous associations between obesity and arthritis [7, 79], it provides additional proof of obesity’s impact on physical functionality [5]. The relationship between elderly obesity and severe arthritis often operates in a vicious cycle: elderly obesity causes limited physical activity and declining energy expenditure, which in turn facilitate progressive weight gain [7, 80]. Our results also show that persons who reported a previous diagnosis with angina and had higher depression scores were also more likely to be diagnosed with arthritis.

Interestingly, there was no significant association between obesity and difficulties with learning new task or recall. In fact, the overweight elderly were less likely to have difficulties with recall or learning new tasks. While the latter finding is counterintuitive, it underscores the importance of early preventive mechanisms in managing midlife overweight conditions to avert transition to obesity, which has been found through preclinical and clinical observations to be associated with mild cognitive impairment in old age [81]. Additionally, the likelihood of severe difficulties with learning a new task or recall was significantly decreased by engagement in vigorous physical activities and having at least secondary education. Therefore, the provision of adult education and the encouragement of physical/aerobic activities among the elderly could be a low-cost strategy to prevent the early onset of potentially more serious chronic conditions.

Although we found no significant association between obesity and ADLs, our results showed that the elderly who grouped as underweight had severe deficiencies with ADLs; i.e. the underweight elderly had difficulties engaging in activities such as standing up from sitting, getting to the toilet, and getting up from lying down. It is possible to attribute this to elderly frailty and sickness. We also found that been diagnosed with angina and reporting unhappiness increased likelihood of having severe deficiencies with ADLs, potentially suggesting that there could be a bidirectional relationship between deficiencies with ADLs and unhappiness.

In terms of IADLs, our results were expected and showed that obesity increased the likelihood of severe deficiencies with IADLs among the elderly by 120%. Thus, obesity created severe difficulties in movement. The obese elderly had difficulties with taking care of household needs, joining community activities, transporting themselves to places, and using public transport. Severe deficiencies in IADLs in this cohort could lead to isolation, which has psychosocial and physical health consequences at the individual level, could restrict their ability to offer any help in the community, and increase their dependency on community services.

Our examination of biosocial and sociocultural factors showed that overweight and obese elderly women had increased expected risks of stage 1 and stage 2 hypertension, a greater likelihood of reporting difficulties with recall and learning new tasks, and deficiencies with ADLs and IADLs. Although our findings are consistent with previous work reporting that chronic conditions are gendered [6, 24, 82], they further demonstrate that the obese elderly women may be exposed to other forms of cardiometabolic diseases than their male counterparts. This is also consistent with recent evidence that hypertension, diabetes, and obesity are more prevalent in women over 50 compared to their male counterparts [83]. These trends call for an urgent need to explore qualitatively the pathways by which women become more susceptible to these health risks in old age.

There are some limitations in this present study worth noting in light of our findings. Due to the cross-sectional nature of the study, we are not able to establish causal effects. A longitudinal study that examines changes in health risks through the life course would add additional rigor to our findings. Also, the wave 2 of the SAGE data we used for this study is yet to be published and made publicly accessible; hence, all outcome variables used are from wave 1 as no other data on Ghana currently exists with such information.

## Conclusions

This paper contributes new knowledge on aging and international health with particular emphasis on elderly obesity and health risks. The paper’s scholarly contribution is four-fold. First, prior studies have focused on the disparate effects of obesity on hypertension, arthritis, functional disability and cognitive impairment, with special emphasis on youth cohorts. This study examines these health risks associated with obesity among the elderly in the context of a developing country. The cultural context of Ghana and the multilevel effects of these health risks make the findings compelling and the policy implications important. Increasing life expectancy and ageing trends call for health policies and planning together to accommodate chronic health conditions for the elderly population. The likely coexistence of elderly obesity with hypertension and other chronic conditions such as arthritis, angina, and diabetes implies attention to chronic conditions among the obese elderly must collectively focus on all possible risk factors.

Second, the factors influencing the transition from stage 1 to stage 2 hypertension have rarely been examined in the existing literature. This paper fills that gap by distinguishing between the risk of stage 1 and stage 2 hypertension, the latter being significant for clinical and medical practice. When diagnosed early, the elderly who are stage 1 hypertensive have the chance to make behavioral changes that could avert future medical complications or early mortality due to stage 2 hypertension.

Third, this study identifies factors such as physical activity that could potentially mediate the effects of obesity on health risks among the elderly. By doing so, our findings underscore the need for nutritional interventions and other prevention strategies to reduce the health risks associated with obesity. This is particularly important now because urbanization and shifts toward more sedentary lifestyles and diets rich in fat, sugar, and animal-based protein in developing nations increase susceptibility to obesity.

Finally, this study points to the need to address the social determinants of health as a critical intervention to ensure the economic and socially disadvantaged in society do not disproportionately carry the burden of these health risks. The multiple health risks associated with obesity among the elderly in Ghana and elsewhere in SSA make it a public health concern that needs to be addressed through the right policies, educational campaigns, and interventions.
